# The impact of age on post-operative outcomes of colorectal cancer patients undergoing surgical treatment

**DOI:** 10.1186/1471-2407-5-153

**Published:** 2005-12-02

**Authors:** Tadas Latkauskas, Giedrė Rudinskaitė, Juozas Kurtinaitis, Rasa Jančiauskienė, Algimantas Tamelis, Žilvinas Saladžinskas, Dainius Pavalkis

**Affiliations:** 1 Unit of Coloproctology, Department of Surgery, Kaunas Medical University Clinics, Eivenių 2, Kaunas, Lithuania; 2 University of Vilnius, Institute of Oncology, Santariskiu 1, Vilnius, Lithuania; 3 Department of oncology, Kaunas Medical University, Eivenių 2, Kaunas, Lithuania

## Abstract

**Background:**

the purpose of study was to evaluate the impact of age on outcomes in colorectal cancer surgery.

**Methods:**

patients on hospital database treated for colorectal cancer during the period 1995 – 2002 were divided into two groups: Group 1 – patients of 75 years or older (n = 154), and Group 2 – those younger than 75 years (n = 532).

**Results:**

In Group 1, for colon cancers, proximal tumors were significantly more common (23% vs. 13.5%, p < 0.05), complicated cases were more frequent (46 % vs. 33%, p = 0.002), bowel obstruction more common at presentation (40% vs. 26.5%, p = 0.001), and more frequent emergency surgery required (24% vs. 14%, p = 0.003). Postoperative overall morbidity was higher in the elderly group, but with no differences in surgical complications rate. Overall 5 year survival was 39% vs. 55% (p = 0.0006) and cancer related 5 year survival was 44% vs. 62% (p = 0.0006). Multivariate Cox analysis showed that age was not an independent risk factor for postoperative mortality.

**Conclusion:**

Preoperative complications and co-morbidities, more advanced disease, and higher postoperative nonsurgical complication rates adversely affect postoperative outcomes after surgery for colorectal cancer in the elderly.

## Background

Colorectal cancer is a disease of elderly, with only 5% recorded in those younger than 40 years. Elderly patients form a highly heterogeneous group in respect of both general physical status, and number and types of co-morbidities [1,2]. This, to some degree, has resulted in different concepts in management, not only from surgical and anesthesiological perspectives, but also from the expectancy of uneventful recovery and long term survival, combined with acceptable quality of life. Historically, it was suggested that elderly patients do not fare well after surgery for colorectal cancer, with high rates of emergency presentations, inoperability and peri-operative mortality [3], although more recent publications have encouraged the same surgical approach as for younger patients [2,4,5]. The purpose of this study was to evaluate impact of age on colorectal cancer presentation, surgical management and outcomes from a single institution serving a population of approximately 2 million over a six year period.

## Methods

Data of all patients treated in Kaunas Medical University Hospital for colorectal cancer during 1996 – 2002 were collected retrospectively and prospectively. Data included: age; gender; location of the tumor; TNM classification; operative risk factors; whether surgery was performed in the emergency or elective settings; incidence of radical or palliative resections; and the short-term and long-term outcomes. Postoperative mortality was defined as death occurring within the first 30 days after operation. All patients were followed up in the outpatient department and were also seen by a consultant oncologist for consideration of adjuvant therapy. A decision to give adjuvant therapy was based on tumour stage, biological age of the patient and co-morbid risk factors. The Lithuanian Cancer Register supplied the date and cause of death of those who died during the follow-up period. Statistical analysis was made using SPSS package. The chi-square or Fisher exact tests were used for comparison of categorical variables between the groups. Cox analysis was used to identify independent factors for postoperative mortality.

## Results

Over the six year period, 686 patients with colorectal cancer were treated. They were divided on the basis of age into two groups: Group 1 included 154 patients (23%) with ages of 75 years and older, and Group 2 included 532 patients (77%), under 75 years of age.

### Demographics (Table 1)

**Table 1:** Age, gender, location of the tumours and TNM classification;

	Group1 (age ≥ 75 years; n = 154)	Group2 (age <75 years; n = 532)	p
Median age	80.7 ± 4.7	58.29 ± 9.9	0,02
Gender			
Male	73	270	0.5
Female	81	262	
Location			
Rectum	65(42%)	212 (39.8%)	0.45
Colon	88 (58%)	319 (60%)	
sigmoid	42 (27%)	170 (32%)	0.24
descendens	3 (2%)	17 (3%)	0.4
left angle	5 (3.2%)	12 (2.2%)	0.5
transversum	2 (1.2%)	48 (9%)	0.001
Right colon:	36 (23%)	72 (13.5%)	0.04
right angle	7 (4.5%)	18 (3%)	0.5
ascendens	19 (14%)	35 (6.5%)	0.02
caecum	10 (6.4%)	19 (4%)	0.1
TNM stage			
T3–4	464 (87%)	129 (83.7%)	0.2
N+	62 (40%)	193 (36%)	0.02
M+	40 (25%)	127 (23%)	0.4

The median age in Group 1 was 80.7 ± 4.7 years, and 58.29 ± 9.9 in Group2. There was no significant difference in gender ratio. There were no differences in ratio of colonic to rectal tumors between the two groups (Group 1: 89 (58%) colonic, 65 (42%) rectal; Group 2: 320 (60%) colonic, 212 (39%) rectal, p > 0.05). However, amongst colonic tumors, proximal lesions (caecum, ascending colon, hepatic flexure and proximal transverse colon) were significantly commoner in Group 1 (36 (23%); Group 2: 72 (13.5%), (p = 0.04).

When compared in respect of tumor stage, the only significant difference lay in nodal status, which was more advanced in Group 1 (node positive disease: Group 1: 62 (40%); Group 2 :193 (36%), (p = 0.02).

### Complications and surgery

Overall preoperative complications (Table 2) were diagnosed in 72 (46%) patients in Group 1, compared with 176 (33%) in Group 2 (p = 0.002), the commonest being bowel obstruction (Group 1: 62 (40%); Group 2: 141 (26.5%), p = 0.001). Co-morbidities were more frequent amongst the elderly patients 80% vs. 55% (p = 0.0001), with more patients in Group 1 classified as ASA 3–5 (p = 0.0001). Emergency surgery (mainly because of bowel obstruction, or perforation) was performed in 115 (17 %) patients overall (Table 2), but was more frequent within the elderly (Group 1: 38(24%); Group 2: 77(14%), p = 0.003).

**Table 2:** Preoperative complications, co-morbidity, ASA (American Society of Anesthesiologists) distribution and procedures performed;

	Group 1 (age ≥ 75 years; n = 154)	Group 2 (age <75 years; n = 532)	p
Preoperative complications:	72 (46%)	176 (33%)	0.002
Obstruction	62 (40%)	141 (26.5%)	0.001
Perforation	8	28	0.47
Co-morbidity	123 (80%)	296 (55%)	0.0001
ASA 3–5	111	248	0.0001
Surgery			
Emergency	38 (24%)	77 (14%)	0.003
Curative	104 (68%)	384 (82%)	0,2
Palliative	50 (32%)	148 (18%)	
Resection rate	131 (85%)	481 (90%)	0,01

On the basis of preoperative staging and surgeon decision concerning radicality, 68% of patients underwent resection with curative intent in group 1, compared with 82% in group 2 (p = 0.2). However, the resection rate was lower in elderly group – 85% vs. 90% (p = 0.01), with palliative colostomy or by-pass surgery performed in 16 (10%) patients in Group 1, compared with 20 (3.7%) in Group 2 (p = 0.0001). There were no other differences between the two groups with regard to the type of operation.

Postoperative complications were recorded in 57(37%) patients in the elderly cohort, compared with 164 (30%) in the younger group (p > 0.05), although general complications (pneumonia, pulmonary failure, pulmonary embolism, arrhythmia, myocardial infarction, cardiovascular failure and urinary infection) were more frequent in the elderly (Group 1: 34 (22%); Group 2: 73 (13.7%), p = 0.02). There were no differences between the groups in surgical complication rate (Group 1: 24 (15.5 %); Group 2: 91 (17%), p = 0.6), either in the elective or emergency setting (Table 3), but the general non-surgical complication rate was higher after elective surgery in the elderly (Group 1: 22 (18.9 %); Group 2: 53 (11.6%), p = 0.04).

**Table 3:** Post-operative complications

	age ≥ 75 years; n = 38; emergency	age <75 years; n = 77; emergency	p	age ≥ 75 years; n = 116; elective	age <75 years; n = 455; elective	p
Overall morbidity	19 (50%)	44 (57.1%)	0,4	38 (32.7%)	120 (26.4%)	0,17
General compl.	12 (31.6%)	20 (25.9%)	0,5	22 (18.96%)	53 (11.6%)	0,04
Surgical compl.	7 (18.4%)	24 (31.2%)	0,14	17 (14.6%)	67 (14.7%)	0,9

### Survival

The postoperative mortality rate was 11% (n = 18) in elderly group, compared with 5% (n = 26) in the younger cohort (p = 0.002). The influence of age, TNM stage, ASA distribution, emergency operation and etc. on postoperative mortality were evaluated using Multivariate Cox analysis. The age was not an independent risk factor for postoperative mortality in mentioned analysis.

**Figure 1:**
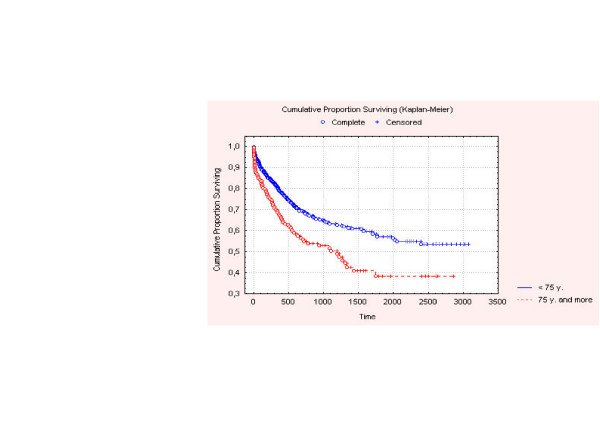
Overall survival of patients.

**Figure 2:**
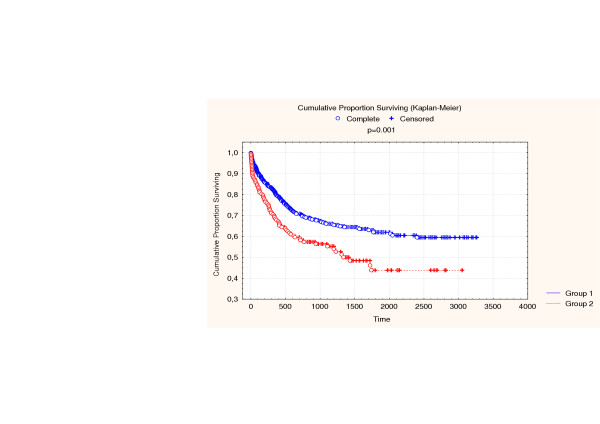
Cancer related survival. (Group 1 < 75 years).

Two year survival (Figure 1) in the elderly group was 55%, compared with 67% in group 2 (p = 0.004). Five year survival was respectively 39% and 55%, (p = 0.0006). Cancer related survival (Figure 2) at 2 years was 59% vs. 70% (p = 0.004), and at 5 years, 44% vs. 62% (p = 0.0006).

## Discussion

No standard definition of "elderly" exists, with different authors using thresholds of 65 [2,6], 70 [7,8,16], 75 [9,10], 80 [2,4] and 85 years [17]. Data from the Lithuanian Office of National Statistics [11] shows that average life expectancy in Lithuania is 71.66 years, 65.88 years for men, and 77.41 years for women. We selected 75 years as the threshold, because it is more than Lithuanian medium life expectancy, and such a division creates a so called elderly group which constitutes approximately one quarter of all colorectal cancer patients, as used in other studies [2]. "Biological" age in different nationalities and populations varies, and it is reasonable that a 75 year old Lithuanian is equivalent to an 80 year old Western European, or 85 year old Japanese, due to differing life expectancies of these populations, although more detailed demographic assessments would be necessary to validate such assumptions.

Previous studies have demonstrated an age-related right shift of colorectal cancer [12,13], this supported by the present study. Marush et al. reported ageing was associated with more locally advanced tumors, but not with metastatic dissemination [2]. This study revealed only differences in nodal status, but no differences between the groups in T stage. However, mechanical bowel obstruction (the most common preoperative complication in both groups), a clinical indicator of locally advanced disease was more frequent in the elderly group. Acute presentation was more frequent in the elderly group, with emergency surgery performed in 24% of such patients, compared to 14% in younger group (p = 0.003), similar to previous studies reported [2,17].

Our study has confirmed that the majority of complications arising in the surgical management of elderly patients with colorectal cancer are not truly surgical, but of a more general nature, both pre- and post-operatively, the latter perhaps compounded by less than optimal preoperative preparation. Menke et al. [14] reported a co-morbidity of 28.1% in patients older than 80 years, and Wolters et al. [8] incidences of 49% for hypertension, 18% for coronary heart disease, and 39% for pulmonary disease, equivalent to our total co-morbidity frequency of 80%, compared to 55% in the younger patient group (p = 0.0001).

Resection rates for elderly patients are usually slightly lower than in younger patients, due to preoperative complications and co-morbidities, and more extensive tumors [2,4,17]. Nevertheless, advances made in surgical technique, anesthetic and postoperative intensive care have resulted in an increase in possibility to perform surgery from 80% up to 95% of cases [16]. The resection rate in our study was 85% in the elderly, comparing with 90% in those younger than 75 years (p = 0.01), with a concomitant increase in the rates of palliative colostomy formation and by-pass procedures in the elderly (10% vs. 3.7%), (p = 0.0001). The recorded reasons for not performing resection included locally advanced disease, presence of preoperative complications and co-morbidities, and emergency nature of the surgery. Postoperative morbidity and mortality is a significant source of concern in the management of the elderly patient with colorectal cancer. Postoperative morbidity is governed by a higher incidence of general complications rate [2,17], with specific surgical postoperative complications occurring at no greater frequency than in younger patients [2]. The difference in rates of general complications following elective surgery, but not after emergency surgery is more difficult to explain.

Overall mortality was 11% in the elderly group, compared with 5% in those under 75 years old (p = 0.002). Emergency surgery for colorectal carcinoma in the elderly is associated with higher morbidity and mortality, reported rates varying between 6% and 38% for emergency operations and 0.9% and 18% for elective operations in those over 70 [7]. The risk of postoperative death in patients over 80 years rises to 11.9 – 38% after emergency surgery, and 7.4 – 11.4% in elective cases [7,8], making the postoperative mortality rates of the present study acceptable.

Overall survival is, not surprisingly, poorer in the elderly, but any differences are much less strong when expressed as cancer related survival rates [4,15]. The overall 2-years and 5-years survival rate in patients over 75 years of age was lower than that observed in younger patients in the present study, perhaps due to the lower frequency of curative operations, and the higher proportion of deaths from other causes in the elderly group. Barrier et al. [4] reported that in those patients operated with curative intent, the 5-year cancer-specific survival rate was not significantly different between the two age groups. Unfortunately the results of the present study do not support this; it is possible however, that a registered cause of death as cancer, without detailed evaluation or postmortem examination, was in fact incorrect.

## Conclusion

In Lithuania, patients with colorectal cancer have similar demographic profiles as those in other countries. Based on a threshold of 75 years, preoperative complication and emergency surgery rates are more common in elderly patients, but postoperative surgical morbidity rates are similar to those observed in younger patients. Postoperative non-surgical morbidity was higher in the elderly group, which influenced postoperative mortality. Overall survival was better in younger patients and the same difference remained in cancer specific long term survival.

## Competing interests

The author(s) declare that they have no competing interests.

## Authors' contributions

TL abstracted data, made data analysis, drafted and revised the manuscript.

GR collected data, performed statistical analysis.

JK participated in data extraction from Lithuanian cancer registry.

RJ collected and abstracted data, participated in the planning of the study.

AT participated in the planning of the study and coordinated the writing of the manuscript.

ZS participated in the planning of the study and coordinated the writing of the manuscript.

DP participated in the planning of the study and coordinated the writing and approved the final manuscript.

All authors read and approved the final manuscript.

## Pre-publication history

The pre-publication history for this paper can be accessed here:



## References

[B1] Ries LAG, Eisner MP, Kosary CL, editors (2000). SEER cancer statistics review, 1973–1997.

[B2] Marusch F, Koch A, Schmidt U, Zippel R, Gastmeier K (2002). Impact of age on the short- term postoperative outcome of patients undergoing surgery for colorectal carcinoma. Colorectal Dis.

[B3] Rankin FW, Johnson CC (1939). Major operations in elderly patients. Surgery.

[B4] Barrier A, Ferro L, Houry S, Lacaine F, Huguire M (2003). Rectal cancer surgery in patients more than 80 years of age. The American J of Surg.

[B5] Sunouchi K, Namiki K, Mori M (2000). How should patients 80 years of age or older with colorectal carcinoma be treated? Long-term and short-term outcome and postoperative cytokine levels. Dis Colon Rectum.

[B6] Paksoy M, Ipek T, Colak T, Cebeci H (1999). Influence of age on prognosis and management of patients with colorectal carcinoma. Eur J Surg.

[B7] Waldron R, Donovan I, Drumm J, Mottram S, Tedman S (1986). Emergency presentation and mortality from colorectal cancer in the elderly. Br J of Surg.

[B8] Wolters U, Isenberg J, Stutzer H (1997). Colorectal carcinoma – aspects of surgery in the elderly. Anticancer Res.

[B9] Tomoda H, Tsujitani S, Furusawa M (1998). Surgery for colorectal cancer in elderly patients- a comparison with younger adult patients. Jpn J Surg.

[B10] Makela J, Kiviniemi H, Laitinen S (2000). Survival after operations for colorectal cancer in patients aged 75 years and over. Eur J Surg.

[B11] Office of national Statistics Demografic and cancer statistics 1996–2000.

[B12] Kempainen M, Raiha I, Rajala T, Souranda L (1993). Characteristics of colorectal cancer in elderly patients. Gerontology.

[B13] Arai T, Takubo K, Sawabe M, Esaki Y (2000). Pathologig characteristics of colorectal cancer in the elderly; a retrospective study of 947 surgical cases. Clin Gastroenterology.

[B14] Menke H, Graf J, Heintz A, Klein A, Junginger T (1993). Risk factors of perioerative morbidity and mortality with special reference to tumor stage, site and age. Zentralbl Chir.

[B15] Edna TH, Bjerkeset T (1998). Colorectal cancer in patients of over 80 years of age. Hepatogastroenterology.

[B16] Poon R, Law W, Chu K, Wong J (1998). Emergency resection and primary anastomosis for left-sided obstructing colorectal carcinoma in the elderly. Br J Surg.

[B17] Colorectal Cancer Collaborative Group (2000). Surgery for colorectal cancer in elderly patients; A systematic review. Colorectal Cancer Collaborative Group. Lancet.

